# Interplay of hypoxia, immune dysregulation, and metabolic stress in pathophysiology of type 1 diabetes

**DOI:** 10.3389/fimmu.2025.1599321

**Published:** 2025-06-04

**Authors:** Rahul Mittal, Joana R. N. Lemos, Prem Chapagain, Khemraj Hirani

**Affiliations:** ^1^ Diabetes Research Institute, University of Miami Miller School of Medicine, Miami, FL, United States; ^2^ Division of Endocrinology, Diabetes, and Metabolism, Department of Medicine, University of Miami Miller School of Medicine, Miami, FL, United States; ^3^ Department of Physics, Florida International University, Miami, FL, United States; ^4^ Biomolecular Sciences Institute, Florida International University, Miami, FL, United States

**Keywords:** hypoxia, type 1 diabetes, immune dysregulation, hypoxia-inducible factors, autoimmune response, regulatory T cells, hyperglycemia, inflammation

## Abstract

Type 1 diabetes (T1D) is an autoimmune disease characterized by the progressive destruction of pancreatic β-cells, leading to insulin deficiency and chronic hyperglycemia. While immune-mediated mechanisms of β-cell destruction are well-recognized, emerging evidence highlights hypoxia as a silent yet critical contributor to T1D pathogenesis. Hypoxia in the pancreatic islets arises from inflammation, vascular dysfunction, hyperglycemia, and immune cell infiltration, creating a microenvironment that exacerbates β-cell dysfunction and amplifies autoimmune responses. Hypoxia-inducible factors (HIFs) play a dual role in regulating adaptive and maladaptive responses to hypoxia, influencing β-cell survival, immune activation, and oxidative stress. Specifically, hypoxia promotes the polarization of macrophages toward a pro-inflammatory M1 phenotype, enhances the differentiation of Th17 cells, and impairs the function of regulatory T cells (Tregs), thereby shifting the immune landscape toward sustained autoimmunity. This perspective discusses the multifaceted role of hypoxia in driving immune dysregulation and β-cell vulnerability in T1D as well as highlights the need for innovative research approaches to target this pathway. We propose future directions that emphasize the development of advanced experimental models to mimic the interplay between hypoxia, hyperglycemia, and immune responses in clinically relevant conditions. Furthermore, we highlight the potential of therapeutic strategies that target hypoxia and its downstream effects to preserve β-cell function and modulate autoimmunity. Collaborative efforts across disciplines will be crucial to translating these insights into clinical innovations that improve outcomes for individuals with T1D.

## Introduction

Type 1 diabetes (T1D) is an autoimmune disease characterized by the progressive destruction of insulin-producing β-cells within the pancreatic islets of Langerhans (1–3). This loss of β-cells results in an inability to produce insulin, a hormone critical for regulating blood glucose levels, necessitating lifelong insulin replacement therapy for affected individuals. While T1D is primarily recognized as an immune-mediated disorder driven by autoreactive T cells targeting β-cell antigens, the microenvironment within the islets plays an increasingly recognized role in modulating disease progression. Emerging evidence highlights hypoxia, a state of insufficient oxygen supply, as a key factor that contributes to β-cell dysfunction (4, 5). It also exacerbates immune activation, forming a vicious cycle that accelerates the destruction of β-cells (6).

Pancreatic islets are among the most vascularized tissues in the body, with a dense capillary network that ensures rapid oxygen and nutrient delivery to β-cells (7). This vascularization is essential for supporting the high metabolic demands of insulin production and secretion (8). However, in T1D, this intricate network is disrupted by multiple factors, including chronic inflammation, hyperglycemia, and immune cell infiltration (9). Inflammatory cytokines such as interleukin-1β (IL-1β) and tumor necrosis factor-alpha (TNF-α) damage endothelial cells as well as impair vascular function, while the recruitment of immune cells to the islets increases local oxygen consumption (10, 11). Hyperglycemia, a hallmark of T1D, further compounds these effects by inducing metabolic stress and promoting vascular dysfunction. Together, these factors create a hypoxic microenvironment within the islets (12).

**Figure 1:**
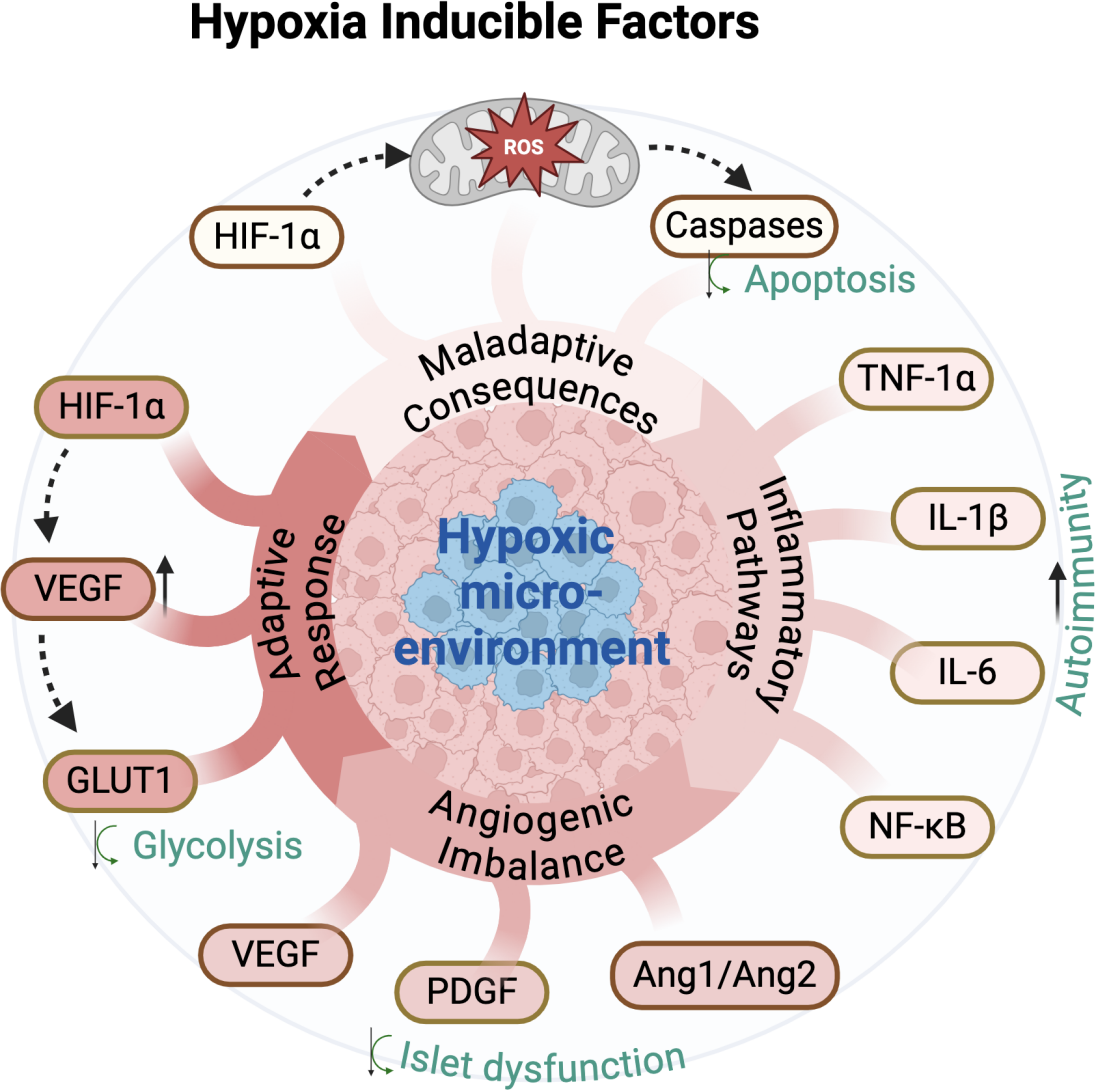
Hypoxia-Induced Pathways: Adaptive and Maladaptive Responses. A schematic representation regarding the role of Hypoxia-Inducible Factors (HIFs) in regulating cellular responses within a hypoxic microenvironment. Hypoxia stabilizes HIF-1α, leading to an adaptive response via upregulation of VEGF and GLUT1, promoting angiogenesis and glycolysis. However, chronic hypoxia induces maladaptive consequences, including ROS production, caspase activation, and apoptosis. Additionally, hypoxia triggers angiogenic imbalance (VEGF, PDGF, Ang1/Ang2) contributing to islet dysfunction. Inflammatory pathways (TNF-1α, IL-1β, IL-6, NF-κB) promote autoimmunity, further exacerbating tissue damage. HIF-1α – Hypoxia-inducible factor 1-alpha; VEGF – Vascular endothelial growth factor; GLUT1 – Glucose transporter 1; PDGF – Platelet-derived growth factor; Ang1/Ang2 – Angiopoietin-1 and Angiopoietin-2; ROS – Reactive oxygen species; TNF-1α – Tumor necrosis factor-alpha; IL-1β – Interleukin-1 beta; IL-6 – Interleukin-6; NF-κB – Nuclear factor-kappa B. Created with BioRender.com

The consequences of hypoxia in T1D extend beyond impaired oxygen supply. Hypoxia directly affects β-cell survival by inducing mitochondrial dysfunction, oxidative stress, and endoplasmic reticulum (ER) stress, all of which compromise insulin secretion and β-cell viability (13, 14) (**Figure 1**). Furthermore, hypoxia triggers maladaptive cellular responses mediated by hypoxia-inducible factors (HIFs), transcription factors that regulate genes involved in angiogenesis, metabolism, and inflammation (15) (**Figure 1**). While transient HIF activation can promote protective mechanisms, such as vascular remodeling and metabolic adaptation, chronic HIF signaling has been implicated in exacerbating β-cell apoptosis and inflammation (16, 17).

In addition to its effects on β-cells, hypoxia significantly influences the immune microenvironment within the islets. Hypoxia-induced metabolic reprogramming of immune cells promotes a shift toward pro-inflammatory phenotypes, including Th17 cells and M1 macrophages, while impairing the function of regulatory T cells (Tregs) that are critical for maintaining immune tolerance (18). This pro-inflammatory milieu perpetuates autoimmune activity and amplifies β-cell destruction. Moreover, hypoxia-induced oxidative stress enhances the release of damage-associated molecular patterns (DAMPs) and the formation of neoantigens, further driving immune activation (19).

Despite advances in understanding the immune mechanisms of T1D, the role of hypoxia as a driver of both β-cell dysfunction and immune dysregulation has been underexplored. Addressing hypoxia in T1D represents a promising therapeutic avenue, as it offers the potential to not only preserve β-cell function but also modulate the autoimmune response. Understanding the role of hypoxia in the pathogenesis of T1D necessitates its integration into established immunopathological frameworks. Hypoxia does not act in isolation but rather potentiates immune dysregulation by intensifying pro-inflammatory cytokine signaling, reprogramming immune cell metabolism toward pathogenic phenotypes, and heightening the susceptibility of pancreatic β-cells to stress-induced apoptosis. This mechanistic interplay highlights the need for a systems biology perspective, wherein hypoxia is recognized not merely as a downstream consequence of islet inflammation, but as an active modulator of both immune and metabolic dysfunction in T1D. By elucidating the interplay between hypoxia, β-cell stress, and immune activation, researchers can identify novel targets for intervention, creating opportunities for therapies that address the underlying mechanisms of T1D and potentially prevent disease onset or progression. This perspective aims to highlight the multifaceted role of hypoxia in T1D and its implications for disease progression and therapeutic innovation. It synthesizes findings from peer-reviewed experimental and clinical literature to integrate current knowledge on hypoxia in T1D pathogenesis. Our analysis draws upon mechanistic studies, animal models, and single-cell transcriptomics to identify converging pathways of β-cell stress and immune dysfunction.

## Sources of hypoxia in T1D

To understand how hypoxia contributes to β-cell failure and immune dysregulation in T1D, it is essential to examine the mechanisms that drive oxygen deprivation within the islet microenvironment. The pancreatic islets are uniquely vulnerable to hypoxia due to their reliance on a dense and highly specialized vascular network that facilitates efficient oxygen delivery to support insulin secretion and metabolic activity. However, in the context of T1D, multiple factors converge to disrupt this delicate balance, leading to the development of a hypoxic microenvironment (**Table 1**).

**Table 1:** Sources of Hypoxia in Type 1 Diabetes (T1D).

Source	Mechanism	Contributing Factors	Impact on Islets
Inflammation and Vascular Dysfunction	Cytokine-induced endothelial dysfunction, increased vascular permeability	Elevated levels of IL-1β, TNF-α, and IFN-γ	Damaged endothelial cells, reduced oxygen delivery, increased islet permeability to immune cells
Hyperglycemia-Induced Stress	Oxidative stress in endothelial cells, increased metabolic demand of beta cells	Prolonged high glucose levels, advanced glycation end-products (AGEs)	Impaired vascular repair, worsened oxygen imbalance, increased beta-cell oxidative stress
Immune Cell Infiltration	High oxygen consumption by metabolically active immune cells (such as macrophages, T cells, B cells)	Autoimmune response, recruitment of pro-inflammatory cells	Oxygen demand surpasses supply, amplifies hypoxia in islets
β-Cell Overactivation	Elevated oxygen consumption due to increased insulin production in response to beta-cell loss	Autoimmune-mediated beta-cell destruction, hyperglycemia	Excessive mitochondrial oxygen utilization, ROS generation, beta-cell stress and dysfunction
Islet Capillary Dysfunction	Loss of pericytes, thickened basement membrane, impaired angiogenesis	Chronic inflammation, vascular damage, endothelial apoptosis	Decreased capillary density, reduced nutrient and oxygen supply
Oxidative Stress	Generation of reactive oxygen species (ROS) that impair cellular metabolism and mitochondrial function	Mitochondrial dysfunction, sustained hyperglycemia	Exacerbation of beta-cell apoptosis and reduced insulin production

### Inflammation and vascular dysfunction

Inflammatory cytokines, particularly IL-1β, TNF-α, and interferon-gamma (IFN-γ), secreted by infiltrating immune cells, not only exert cytotoxic effects on β-cells but also disrupt endothelial cell function within the microvasculature (20). These cytokines impair the structural integrity of islet capillaries by promoting endothelial apoptosis and increasing vascular permeability. The islet endothelium, crucial for maintaining optimal oxygen and nutrient exchange, becomes compromised under inflammatory stress. This leads to increased vascular permeability, reduced nitric oxide bioavailability, and endothelial cell apoptosis. As the vasculature deteriorates, the effective delivery of oxygen and nutrients to β-cells diminishes, contributing to the onset of hypoxia (21). Additionally, chronic inflammation leads to the deposition of extracellular matrix proteins and fibrosis, which physically obstructs oxygen diffusion within the islets (22). This fibrotic remodeling occurs in parallel with increased leukocyte adhesion and transmigration, facilitated by the upregulation of adhesion molecules such as ICAM-1 and VCAM-1 on endothelial surfaces, further perpetuating local inflammation and vascular dysfunction (23). Beyond the islets, systemic vascular dysfunction is frequently observed in individuals with T1D, even in the early stages of the disease. This includes impaired flow-mediated dilation, increased arterial stiffness, and microvascular rarefaction. Such changes are not only markers of early cardiovascular risk but may also reflect persistent low-grade inflammation, endothelial activation, and impaired repair mechanisms. Importantly, alterations in vascular health may precede overt hyperglycemia and serve as early indicators of disease development in at-risk individuals.

### Hyperglycemia-induced stress

Sustained hyperglycemia, a hallmark of T1D, exacerbates hypoxia through several mechanisms. High glucose levels induce oxidative stress in endothelial cells, reducing their ability to respond to angiogenic signals and repair damaged vasculature (24, 25). Furthermore, hyperglycemia increases the metabolic demand of β-cells, forcing them to consume more oxygen for insulin production and secretion (26, 27). This hypermetabolic state creates an oxygen imbalance, where demand surpasses supply, further deepening the hypoxic state. Hyperglycemia also induces advanced glycation end products (AGEs), which contribute to endothelial dysfunction and impair microvascular function (28).

### Immune cell infiltration

The autoimmune nature of T1D results in the infiltration of immune cells, including T cells, B cells, and macrophages, into the pancreatic islets (29, 30). These cells are metabolically active and consume large amounts of oxygen to sustain their pro-inflammatory activity. For example, activated macrophages rely on aerobic glycolysis, a process driven by high oxygen consumption, to produce inflammatory mediators (31–33). Similarly, autoreactive T cells utilize oxygen to proliferate and release cytokines, further straining the already limited oxygen availability within the islets (34). This increased oxygen demand by immune cells compounds the hypoxic burden on β-cells, creating a vicious cycle of oxygen deprivation and immune activation (35, 36).

### β-cell overactivation and metabolic stress

As β-cells are destroyed by autoimmune attack, the remaining β-cells are forced to compensate by increasing their insulin production. This overactivation leads to higher metabolic activity and oxygen consumption. Simultaneously, the stress imposed by hyperglycemia and inflammatory cytokines amplifies oxidative phosphorylation within β-cells, leading to excessive mitochondrial oxygen utilization and the generation of reactive oxygen species (ROS). The combination of high oxygen consumption and ROS-induced mitochondrial dysfunction further exacerbates hypoxia and β-cell vulnerability (37). Unlike other tissues, β cells have relatively low antioxidant capacity, making them particularly susceptible to oxidative stress (38). This environment promotes endoplasmic reticulum stress, unfolded protein response (UPR) activation, and mitochondrial dysfunction, processes that can trigger pro-apoptotic signaling cascades and compromise β-cell viability.

### Islet capillary dysfunction

In T1D, the capillary network within the islets becomes increasingly dysfunctional due to pericyte loss, basement membrane thickening, and impaired angiogenesis. This dysfunction reduces the ability of the capillaries to adequately supply oxygen and nutrients to β-cells. Studies have shown that islet endothelial cells are particularly sensitive to inflammatory and hyperglycemic stress, which accelerates their deterioration and diminishes capillary density over time (39–41).

## Role of hypoxia-inducible factors

Hypoxia-inducible factors (HIFs) are pivotal transcriptional regulators orchestrating cellular responses to oxygen deprivation (42) (**Supplementary Table 1**). Specifically, under normoxic conditions, HIF-1α and HIF-2α undergo prolyl hydroxylation by oxygen-dependent prolyl hydroxylase domain (PHD) enzymes, targeting them for ubiquitination and proteasomal degradation via the von Hippel–Lindau (VHL) E3 ligase (43, 44). In hypoxic conditions, this hydroxylation is inhibited, allowing HIF-α subunits to stabilize, accumulate in the cytoplasm, translocate to the nucleus, dimerize with HIF-1β, and drive the transcription of genes (45). In pancreatic islets, transient HIF activation during early hypoxia confers adaptive advantages. HIF-1α promotes the expression of vascular endothelial growth factor (VEGF), which facilitates angiogenesis to improve oxygen delivery to hypoxic islets (46). Concurrently, HIF-1α enhances the expression of glycolytic enzymes, enabling β-cells and surrounding immune cells to shift from oxidative phosphorylation to glycolysis, thereby supporting cellular energy production and viability despite reduced oxygen availability (47). This metabolic adaptation provides temporary protection to islets under acute hypoxic stress.

However, prolonged HIF activation in chronic hypoxia has maladaptive consequences, severely compromising β-cell function and immune homeostasis (4). Sustained HIF stabilization exacerbates ROS accumulation and oxidative stress, impairing mitochondrial integrity and β-cell insulin secretion. Chronic HIF activity decreases insulin signaling pathways and disrupts the expression of critical insulin-related transcription factors, including PDX1 and MAFA, undermining β-cell identity and functionality (5, 48). Moreover, in immune cells, HIF-1α drives macrophage polarization toward a pro-inflammatory M1 phenotype and enhances the differentiation of Th17 cells, both of which contribute to autoimmune β-cell destruction (49). Simultaneously, HIF-1α diminishes the functionality of regulatory T cells (Tregs) by promoting the proteasomal degradation of FOXP3, a master transcription factor critical for Treg identity and suppressive capacity (50, 51). Additionally, HIF-1α promotes glycolytic metabolism, which antagonizes the oxidative phosphorylation-dependent metabolic program necessary for Treg stability and function (50, 52). This intricate interplay between HIF-mediated immune dysregulation and β-cell dysfunction accelerates the progression of islet pathology (53, 54).

Chronic HIF activation also disrupts angiogenesis within islets, leading to a persistent imbalance. Despite the upregulated VEGF expression, the resultant blood vessels are structurally abnormal, leaky, and non-functional, failing to restore adequate oxygen delivery. This angiogenic dysregulation sustains hypoxia and perpetuates islet dysfunction. Additionally, HIF signaling amplifies inflammatory pathways by promoting the secretion of pro-inflammatory cytokines such as IL-6 and TNF-α from β-cells and infiltrating immune cells. These cytokines exacerbate immune cell recruitment and activation, intensifying islet inflammation. The combined effects of HIF-driven inflammation, oxidative stress, and angiogenic imbalance create a hostile microenvironment that accelerates β-cell apoptosis and disrupts islet homeostasis.

## β-Cell stress pathways: mitochondrial, ER, and autophagic dysfunction

Concurrently with its effects on immune architecture, hypoxia imposes direct cellular stress on pancreatic β-cells. In the subsequent section, we delineate how hypoxia-induced perturbations in mitochondrial function, protein folding, and intracellular degradation pathways contribute to β-cell dysfunction.

### Mitochondrial dysfunction

β-cells exhibit increased sensitivity to mitochondrial dysfunction, particularly when subjected to the simultaneous stressors of hypoxia and hyperglycemia (55, 56). Chronic hypoxia compromises the efficiency of oxidative phosphorylation by limiting oxygen availability, which is a critical substrate for the electron transport chain (57). This results in reduced ATP production and altering cellular energy homeostasis (58). Concurrently, hyperglycemia exacerbates the metabolic burden on mitochondria by driving an excessive flux of glucose-derived substrates through the tricarboxylic acid (TCA) cycle (59). This overloading of mitochondrial metabolism leads to an increased generation of ROS, creating a state of oxidative stress (60). The accumulation of ROS initiates widespread oxidative damage to lipids, proteins, and mitochondrial DNA, impairing the integrity and function of the mitochondria. This oxidative stress also disrupts the delicate balance of intracellular signaling pathways critical for β-cells survival, such as those regulating insulin secretion (56, 61). Additionally, the sustained oxidative environment activates apoptotic pathways, including mitochondrial outer membrane permeabilization and cytochrome c release, which accelerate β-cell death (62).

While mitochondrial dysfunction is well documented in type 2 diabetes, similar mechanisms may contribute to β-cell loss in T1D. However, the direct connection with T1D remains unclear, highlighting the need for future investigations to elucidate the role of mitochondrial impairment in T1D pathogenesis. Emerging evidence suggests that in T1D, immune mediated inflammation driven by cytokines such as IL-1β, TNF-α, and IFN-γ, along with local hypoxia, impairs mitochondrial respiration and elevates ROS production in pancreatic β-cells (63). This oxidative stress disrupts ATP synthesis, damages mitochondrial structures, and activates apoptotic pathways (64–66). Hypoxia further exacerbates mitochondrial dysfunction by stabilizing HIF-1α, which promotes a shift to glycolysis and impairs insulin secretion (4, 67, 68). Additionally, chronic inflammation impairs mitophagy, leading to the accumulation of dysfunctional mitochondria (69–71). These synergistic stressors accelerate β-cell dysfunction and death, highlighting potential therapeutic targets in oxidative stress regulation and immune modulation.

### Endoplasmic reticulum stress

In addition to impairing mitochondrial bioenergetics, hypoxia and hyperglycemia synergistically activate endoplasmic reticulum stress pathways, thereby disrupting insulin biosynthesis and triggering apoptotic signaling cascades. Endoplasmic reticulum (ER) stress occurs when the protein-folding capacity of the ER is overwhelmed, leading to an accumulation of misfolded or unfolded proteins within its lumen. This disruption activates a highly conserved cellular mechanism known as the unfolded protein response (UPR). The UPR is designed to mitigate ER stress by halting general protein translation, increasing the production of molecular chaperones to aid in proper protein folding, and enhancing ER-associated degradation (ERAD) to clear misfolded proteins. However, when ER stress is prolonged or severe, the adaptive mechanisms of the UPR become maladaptive, triggering pathways that promote cell dysfunction and death, primarily through apoptosis (72–74).

In the context of hyperglycemia, elevated glucose levels intensify ER stress. The β-cells are highly sensitive to ER stress due to their essential role in insulin biosynthesis, a process that places a significant burden on the ER. Hyperglycemia increases the demand for insulin production, leading to excessive protein synthesis. This heightened demand exacerbates the likelihood of protein misfolding and impairs the ER’s ability to manage the cellular workload. Consequently, persistent hyperglycemia and ER stress contribute to β-cell dysfunction and eventual apoptosis, undermining insulin production and promoting hyperglycemia in a vicious cycle. Moreover, in hypoxic conditions, often observed in inflamed or poorly vascularized tissues, ER stress is further intensified due to impaired oxygen availability, which disrupts protein folding processes that rely on oxidative environments. Proper formation of disulfide bonds in the ER requires oxygen-dependent enzymes such as ERO1 and protein disulfide isomerase (PDI) (75–78). Hypoxia inhibits these redox reactions, leading to the accumulation of misfolded proinsulin, sustained activation of the unfolded protein response (UPR), and subsequent apoptotic signaling (79, 80). Combined with the effects of hyperglycemia, this dual stressor amplifies UPR activation, leading to chronic inflammation through the release of pro-inflammatory cytokines. This inflammatory state exacerbates systemic metabolic disturbances and cellular damage, contributing to the pathogenesis of T1D (81–84).

### Impaired autophagy

Hypoxia disrupts cellular homeostasis by impairing various metabolic and repair processes, including autophagy (85). Autophagy is a highly conserved cellular mechanism responsible for degrading and recycling damaged organelles, misfolded proteins, and other cytoplasmic debris (86, 87). In pancreatic β-cells, autophagy plays a critical role in maintaining cellular health, especially given their high metabolic activity and demand for protein synthesis to support insulin production. Under normal conditions, autophagy supports β-cell survival by mitigating oxidative stress, removing damaged mitochondria, and preserving mitochondrial quality, which is essential for ATP production and glucose-stimulated insulin secretion (43, 88, 89). During hypoxia, the ability of β-cells to sustain efficient autophagic activity is compromised. Hypoxia directly inhibits autophagic flux by interfering with key steps in the autophagy pathway. Specifically, the low oxygen levels can disrupt the activity of hypoxia-sensitive proteins and signaling pathways, such as AMP-activated protein kinase (AMPK) and mechanistic target of rapamycin (mTOR) (90). AMPK typically promotes autophagy by activating the ULK1 complex, while mTOR acts as a negative regulator of autophagy (91, 92). In hypoxic conditions, dysregulation of these pathways reduces the induction and progression of autophagy, leading to the accumulation of damaged organelles, such as dysfunctional mitochondria. This exacerbates oxidative stress, as impaired mitochondria produce ROS, creating a self-reinforcing cycle of cellular damage (93–95).

Hyperglycemia compounds the autophagic impairment induced by hypoxia. Chronic high glucose levels generate excessive amounts of toxic metabolites, including AGEs and ROS (96). These metabolites further stress cellular systems and increase the demand for autophagic clearance (97–99). However, when autophagic capacity is already compromised due to hypoxia, β-cells are unable to efficiently clear these harmful byproducts. This leads to cellular dysfunction and damage, contributing to β-cell apoptosis and exacerbating the loss of insulin production. Furthermore, hyperglycemia-driven activation of the hexosamine biosynthetic pathway and protein kinase C (PKC) signaling disrupts autophagy by altering post-translational modifications of key autophagy-related proteins (100–102). These modifications reduce the efficiency of autophagosome formation and lysosomal degradation. Additionally, excess glucose exposure causes ER stress, which interacts with and inhibits autophagic pathways, further compounding the inability of β-cells to manage intracellular damage (103).

The combined effects of hypoxia and hyperglycemia create a cellular environment where repair and quality control mechanisms are overwhelmed. The progressive accumulation of damaged organelles, misfolded proteins, and oxidative stress amplifies β-cell dysfunction, contributing to the pathogenesis and progression of diabetes. These findings highlight the critical importance of maintaining autophagic activity for β-cell health and suggest potential therapeutic avenues aimed at restoring autophagic flux in hypoxic and hyperglycemic conditions. Given the multifaceted role of hypoxia in amplifying both immune dysregulation and β-cell intrinsic stress, therapeutic strategies that target hypoxia-adaptive pathways represent a novel and potentially transformative approach for disease modification in T1D.

## Future directions

Recent studies have suggested the potential role of modulating hypoxia in T1D pathogenesis (4, 104–107). A study demonstrated that human umbilical cord-derived mesenchymal stem cells (hUC-MSCs) enhance the survival and function of pancreatic islets under hypoxic conditions via the HIF-1α/PFKFB3 pathway, suggesting a protective mechanism against hypoxia-induced β-cell dysfunction (108). Additionally, research by Kelly et al. revealed that intrauterine growth restriction leads to adaptive and immune responses in fetal sheep islets, including the activation of immune pathways and stress responses, which may predispose individuals to T1D (109). These findings highlight the necessity for advanced experimental models that accurately mimic the hypoxic and immunological milieu of T1D, thereby facilitating the development of targeted therapeutic strategies aimed at preserving β-cell function and modulating autoimmunity. Building on these findings, it becomes increasingly important to elucidate the cellular and molecular mechanisms that mediate the interplay between hypoxia, immune activation, and β-cell stress, thereby guiding the development of precise, mechanism-based interventions.

### Mechanistic studies

Advancing our understanding of the molecular mechanisms linking hyperglycemia, hypoxia, and immune dysregulation in T1D is critical for developing targeted therapeutic strategies. Despite significant progress in characterizing the individual contributions of these factors, the precise cellular and molecular pathways through which they interact remain poorly defined. Future research should aim to dissect how hyperglycemia-induced metabolic stress and hypoxia collectively drive immune activation and β-cell dysfunction.

Organoid platforms that recapitulate the 3D architecture and cellular complexity of human islets are emerging as powerful tools for modeling these interactions under controlled conditions (110). By incorporating endothelial and immune components into islet organoids, researchers can more accurately simulate the hypoxic and inflammatory microenvironment characteristic of early T1D, allowing for longitudinal and mechanistic studies of disease initiation and progression.

Single-cell RNA sequencing (scRNA-seq) can play a pivotal role in elucidating the heterogeneity of cellular responses within the hypoxic islet microenvironment (111, 112). This approach enables the high-resolution mapping of transcriptomic changes in β-cells, endothelial cells, and infiltrating immune cells, revealing dynamic interactions that exacerbate hypoxia and inflammation. Spatial transcriptomics can complement scRNA-seq by providing spatial context to molecular changes, capturing the precise localization of hypoxia-related genes in relation to islet architecture and immune cell infiltration (35, 113, 114). Utilizing these techniques will provide novel insights regarding the role of hypoxia in the pathophysiology of T1D.

### Therapeutic delivery platforms

While advanced *in vitro* and omics-based platforms are critical for decoding the molecular underpinnings of hypoxia in T1D, translating these insights into viable therapies requires parallel innovation in delivery strategies capable of targeting the islet microenvironment with precision. The development of innovative therapeutic delivery platforms offers significant promise in targeting hypoxia and immune dysregulation in T1D. Traditional systemic treatments are often limited by off-target effects and insufficient delivery to the pancreatic islet microenvironment. Encapsulation technologies for β-cell transplants, for instance, could provide localized protection from immune attack while supporting oxygenation and nutrient delivery. These encapsulated transplants can be engineered to enhance survival and function in the hypoxic and inflamed microenvironment characteristic of T1D (115, 116).

Localized drug delivery systems also hold potential for improving therapeutic precision. Nanoparticle-based carriers, for example, can be designed to deliver antioxidants, hypoxia modulators, or anti-inflammatory agents directly to the islet microenvironment, reducing systemic side effects while maximizing efficacy. Hydrogel-based systems that release therapeutic agents in response to specific triggers, such as low oxygen levels or high reactive oxygen species, could offer another level of control. Further exploration of these delivery systems is needed to refine their effectiveness and scalability, as well as to evaluate their long-term safety and durability in clinical trials (117, 118).

### Data-driven approaches

As delivery technologies evolve to address site-specific therapeutic challenges, data-driven approaches offer a complementary framework to optimize intervention timing, predict therapeutic responses, and uncover non-obvious molecular targets within the hypoxia–autoimmunity axis. The integration of data-driven methodologies, particularly machine learning and computational modeling, has the potential to revolutionize the understanding and treatment of hypoxia-related mechanisms in T1D (119, 120). By leveraging machine learning techniques, researchers can extract meaningful insights from complex, large-scale datasets generated from multi-omics analyses, such as transcriptomics, proteomics, and metabolomics, as well as from clinical trials and patient registries. These algorithms can uncover intricate patterns, correlations, and predictive markers that might otherwise remain undetected (121–123). For instance, specific molecular drivers or pathways linking hypoxia to immune dysregulation, oxidative stress, and β-cell dysfunction can be identified, which are critical in the pathogenesis of T1D (124).

In parallel, computational modeling provides a powerful tool to simulate the dynamic and multifaceted islet microenvironment under various physiological and pathological conditions, including hypoxia, hyperglycemia, and inflammatory stress (125). These models enable researchers to explore how β-cells interact with immune cells and their surrounding microenvironment, allowing for the *in silico* testing of hypotheses about disease progression and potential interventions. By incorporating data from experimental studies and clinical observations, computational simulations can predict how changes in oxygen availability, nutrient levels, and inflammatory mediators influence β-cell viability and function (126).

### Developing therapeutic strategies

Building on the foundation of experimental and computational advances, therapeutic efforts now focus on mitigating hypoxia-driven β-cell dysfunction through molecular and tissue-level interventions. Hypoxia in T1D plays a pivotal role in the dysfunction and death of pancreatic β-cells, amplifying the progression of the disease. Therefore, targeting hypoxia will pave the way to develop novel therapeutic modalities for T1D (**Supplementary Table 2**). Restoring oxygen levels in the pancreatic microenvironment could alleviate hypoxic stress and improve β-cell survival (127–129). Oxygenation-based approaches, such as hyperbaric oxygen therapy or oxygen-releasing nanoparticles, have been proposed as potential strategies to counteract hypoxia. By addressing the oxygen supply to β-cells, these strategies may mitigate cellular damage and improve metabolic outcomes. Modulation of HIFs is another promising approach for addressing hypoxia-related β-cell dysfunction. Dysregulated HIF-1α signaling during hypoxia contributes to inflammation, impaired autophagy, and β-cell apoptosis (130). Stabilizing HIF-2α or targeting maladaptive HIF-1α activity could enhance angiogenesis, improve islet vascularization, and support β-cell survival. These strategies highlight the therapeutic potential of manipulating hypoxia signaling pathways to mitigate cellular stress and maintain β-cell health (131, 132).

Islet vascularization is critically impaired in T1D, exacerbating hypoxic conditions and β-cell loss. Promoting angiogenesis through VEGF-based therapies or cell-based approaches, such as mesenchymal stem cells (MSCs), could enhance islet perfusion and oxygen delivery (133, 134). These interventions may also address inflammation-induced vascular damage, improving overall islet resilience and functionality in the context of T1D (135).

Hypoxia-induced autophagy impairment is another significant contributor to β-cell dysfunction in T1D. Autophagy plays a crucial role in removing damaged organelles and maintaining cellular health. Hypoxia disrupts this process, leading to the accumulation of damaged mitochondria, increased oxidative stress, and β-cell death (136, 137). Strategies to restore autophagic flux, such as mTOR inhibition or AMPK activation, could counteract hypoxia-induced cellular damage by reactivating autophagy and enhancing cellular stress resilience (138–140). However, systemic administration of these modulators raises concerns due to their broad physiological effects, including metabolic dysregulation, immune suppression, or unintended impacts on proliferative signaling in non-target tissues (141, 142). To circumvent these risks, localized delivery approaches are being developed to restrict therapeutic activity to the pancreatic microenvironment. These include engineered nanoparticles that exploit local tissue markers or microenvironmental cues (such as pH or hypoxia) for targeted release, oxygen-sensitive or hypoxia-responsive hydrogels that release payloads in response to local oxygen tension and encapsulated islet or beta-cell devices incorporating drug reservoirs or responsive release systems (143–148). Such strategies aim to minimize off-target exposure while ensuring sufficient local concentration to modulate autophagy-regulatory pathways within the islet graft or endogenous pancreas. In islet transplantation, hypoxia remains a major barrier to long-term graft survival and function. Transplanted islets often experience hypoxic conditions due to insufficient vascularization, leading to graft failure. Approaches to mitigate hypoxia, such as preconditioning islets with hypoxia-mimicking agents or incorporating oxygen-releasing materials into encapsulation technologies, have the potential to improve transplant outcomes. Additionally, strategies that reduce hypoxia-induced inflammation and oxidative stress could further enhance graft viability (149).

## Conclusions

The intricate interplay between oxygen availability, immune dynamics, and β-cell resilience in T1D highlights a paradigm shift in our understanding of disease progression. Hypoxia, traditionally regarded as a secondary consequence of inflammatory and metabolic disturbances, emerges as a fundamental driver of β-cell vulnerability. It can act as a molecular amplifier of immune-mediated cytotoxicity, oxidative stress, and metabolic dysfunction, establishing a pathophysiological axis that extends beyond conventional immune-centric frameworks.

Deciphering the hypoxic landscape of pancreatic islets necessitates an integrated, systems-biology approach. The chronic stabilization of HIFs within β-cells and immune infiltrates orchestrates a cascade of transcriptional and metabolic perturbations, precipitating a shift toward maladaptive cellular states (150). This aberrant signaling interface disrupts immune homeostasis, subverts β-cell identity by downregulating essential transcriptional regulators including PDX1, MAFA, and NKX6.1, thereby impairing insulin gene expression and promoting a dedifferentiated or progenitor-like phenotype (151–153). This loss of identity compromises glucose-stimulated insulin secretion and β-cell survival. Consequently, interventions targeting hypoxia-mediated dysregulation represent an unexplored frontier in modifying T1D trajectory.

Advancements in spatial transcriptomics, single-cell metabolic profiling, and *in silico* modeling hold the potential to deconvolute the heterogeneity of hypoxic responses within islet microenvironments. Precision-engineered therapeutic strategies, including hypoxia-responsive biomaterials, oxygenation-modulating nanoformulations, and selective HIF modulation, offer novel avenues for mitigating hypoxia-induced β-cell attrition. Furthermore, the integration of hypoxia-centric interventions with immune-modulatory frameworks may redefine therapeutic paradigms, bridging the gap between metabolic preservation and immunological tolerance.

Although targeting hypoxic signaling in T1D represents a compelling therapeutic avenue, translating these concepts into clinical practice presents considerable complexity. One of the foremost challenges is the ability to selectively modulate oxygen-sensitive pathways within the pancreatic islet microenvironment without perturbing systemic oxygen homeostasis. Pharmacologic agents that influence the activity of hypoxia-inducible factors or related metabolic sensors often exert pleiotropic effects in tissues such as the myocardium, central nervous system, and renal epithelium, where oxygen signaling plays fundamental physiological roles. This raises significant safety concerns, including the potential for disrupting angiogenesis, altering mitochondrial bioenergetics, or impairing adaptive cellular responses under normal oxygen conditions. Additionally, the anatomical and physiological inaccessibility of pancreatic islets complicates the targeted delivery of such interventions. Therapeutic compounds must traverse dense stromal tissue, evade rapid systemic clearance, and retain functional activity within the islet niche, which is often characterized by inflammatory and fibrotic remodeling. Overcoming these delivery and stability barriers necessitates the development of next-generation carrier systems that are both biocompatible and capable of site-specific drug release. To advance these strategies toward clinical utility, it is essential to conduct preclinical assessments in experimental models that accurately recapitulate human islet immunobiology, oxygen gradients, and disease kinetics. Moreover, longitudinal studies evaluating toxicity, pharmacokinetics, immune interactions, and off-target responses are critical for establishing a robust safety profile. Collectively, these efforts are foundational to ensuring that hypoxia-focused interventions are both biologically viable and clinically translatable in the context of T1D.

A comprehensive elucidation of the molecular interfaces between oxygen deprivation, β-cell stress responses, and immune-mediated cytotoxicity is essential for the rational development of targeted therapeutic interventions in T1D. This evolving paradigm positions hypoxia not solely as a passive biomarker of disease progression, but as an actionable pathogenic determinant amenable to intervention. By harnessing emerging insights into hypoxia-induced signaling cascades and their impact on β-cell function and immune dynamics, future therapeutic strategies may be optimized to preserve β-cell identity, suppress autoreactive immune effector mechanisms, and fundamentally alter the disease trajectory toward sustained glycemic control and immune tolerance.

## Data Availability

The original contributions presented in the study are included in the article/**Supplementary Material**. Further inquiries can be directed to the corresponding authors.
