# Supplementary material for: Interplay of hypoxia, immune dysregulation, and metabolic stress in pathophysiology of type 1 diabetes
Source: Front Immunol. 2025 Jun 4;16:1599321. doi: 10.3389/fimmu.2025.1599321 (PMC12174378; doi:10.3389/fimmu.2025.1599321)
Supplement: Supplementary file 1 [file DataSheet1.pdf]

Supplementary Table 1: Role of Hypoxia-Inducible Factors in Pancreatic β-Cell Survival and Dysfunction

Role	Mechanism	Effects	Key Molecules Involved	Implications in Diabetes
**Adaptive Response**	- HIF-1α activation under low oxygen.<br>- Upregulates VEGF, promoting angiogenesis.<br>- Shifts metabolism to GLUT1-mediated glycolysis.	- Temporary beta-cell survival.<br>- Maintains ATP production via glycolysis.<br>- Reduces ROS in early hypoxia.	HIF-1α; VEGF; GLUT1	- Short-term adaptation to hypoxia.<br>- Beneficial in islet transplantation.
**Maladaptive Consequences**	- Chronic HIF-1α activation leads to ROS accumulation.<br>- Mitochondrial dysfunction and Caspases-mediated apoptosis.	- Impaired insulin secretion.<br>- Increased immune dysregulation.<br>- Progressive beta-cell failure.	HIF-1α; ROS; Caspases	- Leads to beta-cell exhaustion.<br>- Contributes to disease progression.
**Angiogenic Imbalance**	- VEGF dysregulation leads to leaky, immature capillaries.<br>- Persistent hypoxia and islet dysfunction.	- Worsens oxygen supply.<br>- Increases beta-cell stress and dysfunction.	VEGF; PDGF; Ang1/Ang2	- Aggravates islet dysfunction in T2D.<br>- Potential target for therapy.
**Inflammatory Pathways**	- Hypoxia induces TNF-α, IL-1β, IL-6, recruiting immune cells.<br>- Activates NF-κB, promoting inflammation.	- Chronic inflammation.<br>- Accelerated beta-cell destruction.<br>- Increased autoimmunity.	TNF-α; IL-1β; IL-6; NF-κB	- Drives beta-cell loss in T1D.

HIF-1α – Hypoxia-inducible factor 1-alpha; VEGF – Vascular endothelial growth factor; GLUT1 – Glucose transporter 1; ROS – Reactive oxygen species; Caspases – Enzymes involved in programmed cell death (apoptosis); PDGF – Platelet-derived growth factor; NF-κB – Nuclear factor kappa-light-chain-enhancer of activated B cells; TNF-α – Tumor necrosis factor-alpha; IL-1β – Interleukin-1 beta; IL-6 – Interleukin-6.

Supplementary Table 2: Therapeutic Strategies Targeting Hypoxia in Type 1 Diabetes

**Approach**	**Mechanism**	**Potential Benefit**
HIF Modulators	Target chronic HIF stabilization	Reduce beta-cell apoptosis and inflammation
Antioxidants	Scavenge ROS and reduce oxidative stress	Alleviate mitochondrial dysfunction and beta-cell stress
Pro-Angiogenic Therapies	Promote functional angiogenesis and oxygenation	Enhance islet vascularization and oxygen delivery
Encapsulation Technologies	Oxygen-permeable materials to protect transplanted beta cells	Improved survival and function of beta-cell transplants
Nanoparticle-Based Drug Delivery	Targeted delivery of hypoxia modulators and anti-inflammatory agents	Minimize systemic side effects, maximize local efficacy
